# Mantis shrimp identify an object by its shape rather than its color during visual recognition

**DOI:** 10.1242/jeb.242256

**Published:** 2021-04-23

**Authors:** Rickesh N. Patel, Veniamin Khil, Laylo Abdurahmonova, Holland Driscoll, Sarina Patel, Olivia Pettyjohn-Robin, Ahmad Shah, Tamar Goldwasser, Benjamin Sparklin, Thomas W. Cronin

**Affiliations:** University of Maryland, Baltimore County, UMBC Department of Biological Sciences, 1000 Hilltop Circle, Baltimore, MD 21250, USA

**Keywords:** Object recognition, Learning, Memory, Ethology, Pavlovian conditioning, Animal behavior, Stomatopod, Marine biology, Visual guidance

## Abstract

Mantis shrimp commonly inhabit seafloor environments with an abundance of visual features including conspecifics, predators, prey and landmarks used for navigation. Although these animals are capable of discriminating color and polarization, it is unknown what specific attributes of a visual object are important during recognition. Here, we show that mantis shrimp of the species *Neogonodactylus oerstedii* are able to learn the shape of a trained target. Further, when the shape and color of a target that they had been trained to identify were placed in conflict, *N. oerstedii* tended to choose the target of the trained shape over the target of the trained color. Thus, we conclude that the shape of the target was more salient than its color during recognition by *N. oerstedii*, suggesting that the shapes of objects, such as landmarks or other animals, are important for their identification by the species.

## Abstract (precis)

**Summary:** Mantis shrimp can identify an object by its shape and seem to use the shape of an object over its color when recognizing it.

## INTRODUCTION

Each species of animal living in a given space experiences its own distinct sensory world, known as its ‘umwelt’ (von Uexküll, 1957). The sensory structures responsible for an animal's perception of its environment are metabolically taxing tissues that are often under strong selection pressures to permit the recognition of biologically relevant stimuli, while ignoring much of the available information an environment has to offer. Despite their complexity, the visual systems of stomatopod crustaceans are likely to follow this generalization. Better known as mantis shrimp, these animals are renowned for their visual systems, which in most species enable spatial and motion vision (Cronin et al., 1988; Marshall and Land, 1993); color and multispectral UV vision, with some species exhibiting photoreceptor spectral sensitivities ranging from the deep-UV to far-red wavelength ranges (300–720 nm; Cronin and Marshall, 1989a,b; Cronin et al., 1994, 2014a); and linear and circular polarization receptivity (Marshall et al., 1999; Chiou et al., 2008). The compound eyes of many stomatopod species have a relatively high visual acuity; for instance, *Gonodactylus chiragra*, an animal typically about 8 cm in length, achieves a resolution of 0.8 cycles deg^–1^ (Marshall and Land, 1993). The ability of stomatopods to learn novel visual stimuli has been previously demonstrated with color, linear polarization and circular polarization cues (Marshall et al., 1996, 1999; Chiou et al., 2008; Thoen et al., 2014). Taken together, it is clear that visual information is an important part of a stomatopod's sensory experience and is likely critical for its survival.

Mantis shrimp mostly reside in shallow tropical marine waters worldwide. These locations offer some of the most structurally complex and colorful environments on Earth, and therefore contain many visual features. In these environments, mantis shrimp typically occupy small holes or crevices in the marine substrate for use as burrows, where they reside concealed for most of the day. Mantis shrimp consume a variety of prey (deVries et al., 2016), many of which are brightly colored, and they use colored signals to communicate with one another (Caldwell and Dingle, 1975; Hazlett, 1979; Cheroske et al., 2009; Chiou et al., 2011; Franklin et al., 2019). Furthermore, mantis shrimp of the species *Neogonodactylus oerstedii* exhibit impressive navigational abilities when returning to their burrows from foraging excursions. These animals use landmarks, if available, in parallel with path integration to quickly pinpoint the location of their burrows (Patel and Cronin, 2020a,b,c). The benthic habitats *N. oerstedii* occupy are abundant with potential visually informative features including sponges, coral, rock and aquatic vegetation: structures of distinct shapes and colors.

Because color may be informative in many aspects of a mantis shrimp's life, and because these animals use landmarks for navigation when available, this raises the question of what qualities of an object are evaluated by mantis shrimp during recognition. Considering that mantis shrimp have reasonably acute visual systems and are known to possess color vision, we were interested in determining whether *N. oerstedii* learns to recognize a visual target using its shape and/or its color.

## MATERIALS AND METHODS

### Animal care

Individual *Neogonodactylus oerstedii* (Hansen 1895) collected in the Florida Keys, USA, were shipped to the University of Maryland Baltimore County (UMBC). Animals were housed individually in 30 parts per thousand (ppt) seawater at room temperature under a 12 h:12 h light:dark cycle. Animals were fed whiteleg shrimp, *Litopenaeus vannamei*, once per week when food was not acquired during training sessions. Seventy-eight individuals (31 males and 47 females) that survived over 4 weeks in captivity were used for the study. Testing data were collected from 20 individuals (8 males and 12 females). All individuals were between 30 and 70 mm long from the rostrum to the tip of the telson.

### Experimental apparatus

**Fig. 1.**
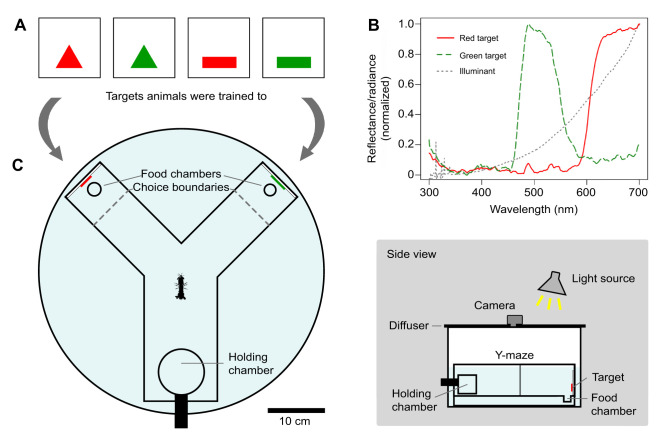
**Layout of experimental setup.** (A) The four targets used during the experiment: a red triangle, a green triangle, a red rectangle and a green rectangle. (B) Averaged normalized reflectance spectra (300 to 700 nm) of the red targets (solid red line) and green targets (dashed green line) and normalized radiance spectrum of the light source (dotted gray line). (C) A Y-maze was placed in a cylindrical tank with an incandescent light source centered above it. A diffusing filter was placed above the arena. The filter had a centered hole, where the lens of a camera was fitted to record each trial. The Y-maze contained an entrance arm and two choice arms oriented 90 deg from one another. A cylindrical holding chamber was centered at the end of the entrance arm. At the end of each choice arm laid a hole set below the floor of the Y-maze. A food reward could have been placed in either hole. One of the targets in A was placed at the end of each choice arm as indicated. The dashed lines in the choice arms of the arena indicate the point at which an animal was recorded as having made a choice.

A Y-maze consisting of an entrance arm and two choice arms oriented 90 deg from one another was constructed out of white acrylic sheets (Fig. 1C). The end of each arm of the Y-maze had a hole in the floor, hidden when viewed from a distance. A food reward was placed in either of these holes. The Y-maze was placed in a cylindrical tank with an incandescent light source (Sylvania SPOT-GRO^®^ 65W) centered above it (the normalized radiance spectrum of the light source is plotted in Fig. 1B). A diffusing filter was positioned on the top of arena below the light source. The filter had a centered hole, where the lens of a small video camera was fit to record each trial. Trials were observed from the screen of this camera. Flat targets made of colored, transparent plastic cemented on a solid white background were placed at the end of each choice arm. Four targets were used during the experiment: a red rectangle, a green rectangle, a red triangle and a green triangle (Fig. 1A,B). Because stomatopods in previous behavioral experiments successfully learned to discriminate red and green colored targets (Marshall et al., 1996), targets of these colors were chosen for the present study. The rectangle and triangle had an angular size (width×height) of 12×4 deg and 9.3×7.8 deg when viewed from the entrance to the choice arm, respectively. A cylindrical holding chamber was centered at the far end of the entrance arm. The holding chamber was designed to be rotated on its side by a researcher, allowing an animal placed inside the chamber access to the rest of the Y-maze.

### Spectrometry

Reflectance measurements of the colored targets were taken in a dark room using an Ocean Optics USB2000 spectrometer connected to a 3 m long, 400 µm diameter, fiber-optic cable. Reflectances were measured from 300 to 700 nm relative to a ‘Spectralon’ white standard using a PX-2 pulsed xenon light source. Radiance measurements of the light source were taken using the same spectrometer.

### Experimental procedures

#### Training

Each *N. oerstedii* individual was randomly assigned to be trained to one of the four target color and shape combinations described above. During training trials, the focal target (e.g. red triangle) was placed at the end of a randomly chosen arm with food in the chamber at its end as a reward. The target of opposite shape and color (e.g. green rectangle) was placed without food at the end of the other arm. A stomatopod was placed in the holding chamber before a trial and allowed 5 min to adjust to its surroundings. After this time, the holding chamber was turned, allowing the animal to enter the arena, initiating the experiment. Once a stomatopod entered the arena, the first choice arm it traveled down was noted once it passed the choice boundary of the arm, at two-thirds of the length of the arm. If it found the food, the experimental animal was allowed 5 min to eat as a reward before being removed from the arena. If the food was not found within 10 min, the animal was removed from the arena. Each animal experienced the training procedure twice per week. After each individual training session, the water in the arena was mixed to prevent olfactory cues from influencing the choice of subsequent training sessions.

At the end of each week, the percentage of correct choices each individual made since the start of training was calculated. Individuals entered the testing phase when they had made a correct choice 80% (or greater) of the time during training trials over the previous 4 weeks, in combination with having found the food 50% (or greater) during that time. Individuals were required to have been trained for at least 1 month (eight training trials) before being considered for testing.

#### Testing

The procedure of the testing phases was identical to that of the training phase except that no food reward was offered during testing sessions. Trained stomatopods were subjected to three distinct tests: (1) a shape recognition test, (2) a color recognition test and (3) a conflicting cues test (see Fig. 3). Initially, only the conflicting cues test was conducted. Once it became clear that animals would indeed perform well in the conflicting cues test, we continued testing using all three test types. At this time, individuals experienced these three types of tests in a randomized order. Once testing began, training or testing occurred twice per week with two training sessions administered between each testing session to facilitate reward seeking between tests.

##### The shape recognition test

In order to test whether *N. oerstedii* could distinguish the shape of the trained target, the cue of the same shape and color as that to which the individual had been trained was placed at the end of one arm of the Y-maze (e.g. red triangle). The cue of the opposite shape and the same color of that to which the individual had been trained was placed at the end of the other arm (e.g. red rectangle). A correct choice was recorded if the stomatopod chose the arm displaying the cue with the trained color and shape.

##### The color recognition test

In order to test whether *N. oerstedii* could distinguish the color of the trained target under the conditions of our study, the cue of the same shape and color as that to which the individual had been trained was placed at the end of one arm of the Y-maze (e.g. red triangle). The cue of the same shape and the opposite color was placed at the end of the other arm (e.g. green triangle). A correct choice was recorded if the stomatopod chose the arm displaying the cue with the trained color and shape.

##### The conflicting cues test

In order to test whether *N. oerstedii* relied more on the shape or color of a target when recognizing it, the cue of the same shape and opposite color as that to which the individual had been trained was placed at the end of one arm of the Y-maze (e.g. green triangle). The cue of the opposite shape and the same color was placed at the end of the other arm (e.g. red rectangle). Neither cue was of the shape and color combination identical to the one which the animal was trained to recognize. As there was no ‘correct’ choice in this test, the color and shape of the selected target was recorded for each trial in which the animal made a choice.

### Statistical analyses

All statistical analyses were run in R (v3.3.1, https://www.r-project.org/) with the ‘car’, ‘glmer’, ‘lme4’ and ‘effectsize’ plugins.

Generalized linear mixed modeling (GLMM) was used to analyze the data for each of the three tests. Our models used animal choices during testing as the variable of interest, specifying a binomial error distribution (link function ‘logit’). Because individual stomatopods were tested more than once, the models for each test included individual ID as a random term. As we used both males and females for our study, sex was also included as a random term for our full models; however, because sex did not significantly increase the explanatory power of our models, it was removed from our final models. Individual ID did not significantly increase the explanatory power of our models, but was left in the final models to account for repeated measures. Effect sizes, reported as Cohen's *D* values, were calculated from the *Z*-score outputs of the GLMMs. All statistical outcomes are reported in Table S1.

## RESULTS

### *Neogonodactylus oerstedii* learned to identify a specific visual target over time

**Fig. 2.**
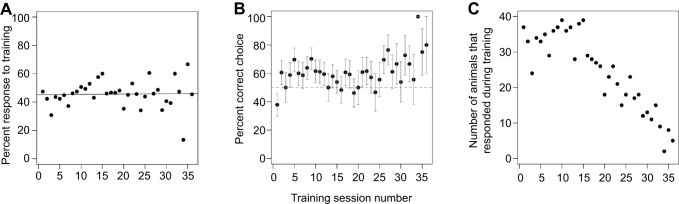
**Training results.** (A) *Neogonodactylus oerstedii* located food during the training procedure approximately half of the time. The solid line represents the line of best fit. (B) *Neogonodactylus oerstedii* associated food with their respective trained targets over time. Each point represents the percentage of animals that correctly chose the target they were training to from all animals who made choices during that training session. Error bars represent ±s.e.m. The dashed line (at 50%) marks represents a random choice proportion. (C) Sample size per point in B. The number of animals undergoing training decreased over time because animals either progressed to the testing procedure or died during the course of the study.

*Neogonodactylus oerstedii* individuals (*n*=78) were trained to one of four targets of a specific color and shape combination (either a red rectangle, red triangle, green rectangle or green triangle) using a paired food reward in a dichotomous choice Y-maze (Fig. 1). On average, animals responded to the paradigm (i.e. made a choice) approximately half of the time (Fig. 2A). From these choices, animals learned to associate food with their respective trained targets over time (Fig. 2B). Of the 78 stomatopods that were trained, 20 individuals reached the criteria set to progress to the testing procedure (see Materials & Methods for criteria). Training was successful for animals trained to all possible target color and shape combinations (Table S2).

### *Neogonodactylus oerstedii* recognized the trained target by its shape, not its color

Once animals reached the performance criteria to enter the testing phase, they were tested in three separate procedures: a shape recognition test, a color recognition test and a conflicting cues test.

**Fig. 3.**
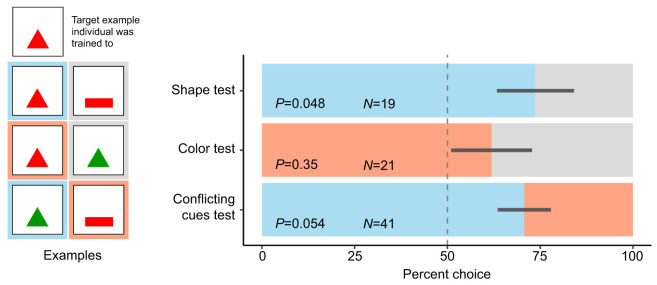
***Neogonodactylus oerstedii* recognized the target by its shape, not its color.** Blue and red bars represent proportions of choices during testing that were for the target of the correct shape and color, respectively. Grey bars represent proportions of choices during testing that were for the incorrect target. Dark grey lines represent standard errors of the means. The vertical dashed line marks a 50% proportion of choices (i.e. a random choice proportion). Examples of targets placed in either arm of each test for an individual that was trained to associate food with a red triangle are found on the left of each bar.

During the shape recognition test, both arms contained targets of the color to which an animal had been trained, but the target in each arm was of a different shape. Stomatopods significantly chose the arm with the shape to which they had been trained, indicating that they recognized the shape of their trained target (*P*=0.048, *Z*=1.976, Cohen's *D*=0.91, *N*=19; Fig. 3, Table S1).

During the color recognition test, both arms contained targets of the shape they were trained to but the color of the target differed per arm. During this task, stomatopods more often than not chose the arm with the same color target as the one to which they had been trained; however, this relationship was not significantly different from a random choice distribution (*P*=0.35, *Z*=0.934, Cohen's *D*=0.41, *N*=21; Fig. 3, Table S1).

During the conflicting cues test, one arm contained a target with the same shape but opposite color to the target to which they were trained, while the other arm had a target with the same color but alternate shape to the trained target. In this situation, individuals tended to choose the arm with the trained shape over the arm with the trained color (*P*=0.054, *Z*=1.927, Cohen's *D*=0.88, *N*=41; Fig. 3, Table S1). This result reflected the results of the shape and color discrimination tests, implying that the shape of the trained target was more important than the target's color to *N. oerstedii* during recognition in our task.

Choices made during each test were not influenced by the shape or color of the trained target (Table S3).

## DISCUSSION

Our study demonstrates that mantis shrimp are able to recognize objects of distinct shapes. We found that mantis shrimp tended to recognize the trained object by its shape rather than its color (Fig. 3). Because mantis shrimp use landmarks during navigation (Patel and Cronin, 2020c), the findings in our study suggest that the shape of a landmark may be more important than its color when being identified by a mantis shrimp during navigation. Similarly, the shapes of prey, predators and body structures used for signaling may be useful for recognition and for generating appropriate behavioral responses.

Identifying an object by its shape might be more effective than recognizing its color when the object is viewed underwater. In water, contrast attenuates with distance and depth owing to the absorption and scattering of light. This is especially true for color information underwater, where the spectral range of incoming daylight or of an object's color is rapidly trimmed to primarily blue light with increasing distance and depth (Cronin et al., 2014b). Because of this, achromatic cues are generally more reliable than color appearance in water, as the colors of objects vary with the distance and the depth of viewing, while their shapes remain unchanged. Therefore, the shapes of objects may be more reliable cues to their identity than their colors when viewed by mantis shrimp in ecologically relevant situations.

The edges of objects are important for recognition by many animals, including humans (Shapley and Tolhurst, 1973) and honeybees (Lehrer et al., 1990), so it is reasonable to hypothesize that mantis shrimp do the same. Shape recognition is likely to be critically important to mantis shrimp when they are recognizing landmarks, which they use to locate their home burrow during navigation (Patel and Cronin, 2020c). In other arthropods, landmark navigation involves retinal image matching, where the field of view seen while navigating is matched to a stored retinal ‘snapshot’ of the view of their goal (Cartwright and Collett, 1983; Akesson and Wehner, 2007). During these tasks, the edges of landmarks appear to be important for image matching and distance estimations (Cartwright and Collett, 1983; Harris et al., 2007). Therefore, edge detection of objects may be critical during navigation as well as for other aspects of a mantis shrimp's life, such as signal recognition, food identification and recognition of predatory threats.

In our study, mantis shrimp failed to learn the colors of the targets to which they were trained. This may have been due to some aspect of our experimental design that did not favor the learning of color information. Conditioning experiments with other animals have demonstrated that multiple redundant cues can compete during associative learning, allowing one cue to overshadow the learning of another one; for example, honeybees trained to a combined color and odor stimulus were unable to learn the color to which they were trained, despite the fact that color is easily learned by honeybees when they are trained to a colored stimulus in isolation (Couvillon et al., 1983; Menzel, 1990). In our experiments, we combined shape and color during associative learning. The apparent failure of our experimental animals to choose a target on the basis of color suggests that shape was a more relevant cue in the task we gave the mantis shrimp, and therefore may have overshadowed the learning of the color of the target. Mantis shrimp can learn to recognize and discriminate color in other circumstances (Marshall et al., 1996). Therefore, when shapes are similar (as in the tests employed by Marshall et al., 1996), color may become more important in discriminating them.

Owing to the radiance distribution of the light source used during our experiments, red targets were better illuminated than green targets (Fig. 1B). However, the red-sensitive photoreceptors in the midband of the stomatopod eye (a region specialized for color vision as well as for some polarization vision modalities) have far less than half the sensitivity of the green color channels due to the anatomical tiering of filters and photoreceptors in the region (Cronin et al., 1994). Also, the main rhabdomeric photoreceptors in the proximal hemispheres of the eye (likely achromatic channels where relative brightness might actually be evaluated) are much more sensitive in the green than the red wavelength ranges (Cronin and Marshall, 1989a,b). Therefore, in the eyes of *N. oerstedii*, the green targets used during our experiments may have appeared brighter than the red targets, despite the red targets being better illuminated. All this taken into account, stomatopods may have, in principle, observed brightness as well as color differences between the presented targets, complicating the color vision task. However, Marshall et al. (1996) found that stomatopods could not learn the relative brightness of grey targets, but that they were able to identify red and green targets in the presence of both bright and dark grey ones, suggesting that color cues may have been more salient than brightness cues to mantis shrimp in our experiment as well.

Most mantis shrimp possess fabulously elaborate color vison systems. Although color did not seem to be critical for object recognition in this study, mantis shrimp are likely to favor color discrimination for other specific tasks. Many mantis shrimp have colorful body surfaces, some of which are used for signaling (Caldwell and Dingle, 1975; Hazlett, 1979; Cheroske et al., 2009; Chiou et al., 2011; Franklin et al., 2019). Owing to mantis shrimps' powerful weaponry and aggressive territoriality, signaling intent may be an important way to circumvent a potentially fatal encounter. Many mantis shrimp species possess colorful signals on the inner sides of their raptorial appendages, termed meral spots. The colors of these spots often are distinct in coexisting species. Because multiple stomatopod species are often found occupying the same reef patches, the color of signals such as these meral spots might be useful for species recognition when identifying conspecifics. Color vision might also have other functions for mantis shrimp, such as contrast enhancement when hunting and/or avoiding predators at shallow depths (Cronin et al., 2014c). Carl von Hess (1913) and Karl von Frisch (1914), early researchers studying color vision in honeybees, disagreed about the abilities of these animals to discriminate color [even though across the Atlantic, experimentation by Charles Turner (1910) suggested that honeybees possessed color vision, though the work was not definitive]. The disagreement arose because the researchers chose different behavioral contexts in their studies. We now know that bees use color for nest and flower identification (the contexts in which Turner and von Frisch tested color vision), not for escape runs toward light (von Hess's approach; see Menzel and Backhaus, 1989). Similarly, a mantis shrimp's reliance on color vision surely differs depending on the contextually varied situations it encounters.
